# Is MIStreatment of women during facility-based childbirth an independent risk factor for POstpartum Depression in Ethiopia and Guinea? A mixed methods prospective study protocol—MISPOD study

**DOI:** 10.1186/s12978-024-01850-w

**Published:** 2024-09-04

**Authors:** Anteneh Asefa, Samson Gebremedhin, Alexandre Delamou, Bruno Marchal, Lenka Benová

**Affiliations:** 1 Department of Public Health, Institute of Tropical Medicine, Antwerp, Belgium; 2 School of Public Health, Addis Ababa University, Addis Ababa, Ethiopia; 3 Centre National de Formation Et de Recherche en Santé Rurale, Maférinyah, Forécariah Guinea; 4 Africa Center of Excellence, University Gamal Abdel Nasser, Conakry, Guinea; 5 Centre d’Excellence d’Afrique Pour la Prévention et le Contrôle des Maladies Transmissibles (CEA-PCMT), Université Gamal Abdel Nasser de Conakry, Conakry, Guinea; 6 Centre National de Formation et de Recherche en Santé Rurale de Maferinyah, Forecariah, Guinea

**Keywords:** Postpartum depression, Mistreatment (disrespect and abuse), Mental health, Ethiopia, Guinea

## Abstract

**Background:**

Worldwide, 10% of postpartum women experience postpartum depression, which can lead to diverse sequalae at individual, family, and societal levels. In sub-Saharan Africa, it is estimated that 17% of women experience depression in the postpartum period, which could be an underestimate as 48% of women in the region do not receive postnatal care (81% in Ethiopia and 51% in Guinea) and a large share of postpartum depression remains undiagnosed and untreated as a result. Globally, despite a critical evidence gap, there are growing reports of postpartum depression among women mistreated (disrespected and abused) during childbirth in health facilities, making a strong case to examine the association between mistreatment and postpartum depression. This study in Addis Ababa (Ethiopia) and Conakry (Guinea) uses a mixed methods design to 1) examine the link between mistreatment and postpartum depression, 2) explore the health system capacity to provide respectful maternity care and maternal mental health services, and 3) explore the experiences of women in accessing care and support for postpartum depression.

**Methods:**

We will conduct a prospective longitudinal survey of women (434 in Addis Ababa and 408 in Conakry) from the third trimester of pregnancy to eight weeks postpartum and carry out in-depth interviews with key health system informants (20–25 in each city) and women who recovered from a clinically confirmed episode of postpartum depression (15–25 in each city). Quantitative data from the women’s survey will be analysed using a multilevel mixed-effects model; qualitative data from key-informants will be analysed by using a hybrid thematic analysis approach, whereas data from women’s in-depth interviews will be analysed using the phenomenological approach. The inclusion of two different settings in our study (Addis Ababa and Conakry) will enable us to apply a comparative health systems lens to explore the dynamics of respectful maternity care and maternal mental health services within the broader health systems of the two countries (Ethiopia and Guinea).

**Discussion:**

The findings from this study will inform actions aimed at mitigating the mistreatment of women in maternity settings and improving promotive, preventive, and treatment interventions for postpartum depression in Ethiopia and Guinea. The findings can also be extrapolated to other low-resource settings.

**Supplementary Information:**

The online version contains supplementary material available at 10.1186/s12978-024-01850-w.

## Background

The burden of mental health conditions is growing globally challenging the achievement of international development goals in addition to their multidimensional negative impacts at individual, family, and societal levels [1]. According to the estimates of the Global Burden of Disease Study, mental disorders accounted for 14.6% of years lived with disability and 4.9% of disability-adjusted life years, globally [2]. Postpartum depression constitutes a significant share of those mental disorders despite the availability of cost-effective preventive, screening, and treatment approaches [3, 4]. Such gaps in the detection and treatment of postpartum depression contribute significantly to the worsening of preventable consequences of the disorder, including poor mother-baby attachment, individual harm, and impaired child growth and development [3]. Existing evidence shows that life stress, pregnancy and childbirth complications, unwanted pregnancy, infant loss, dissatisfaction with marital relationship, spousal violence, lack of social support, and history of depression are risk factors for postpartum depression [5].

In sub-Saharan Africa, it is estimated that 17% of postpartum women experience postpartum depression (compared to 10% globally) [6]. This may be an underestimate as 48% of women in the region do not receive postnatal care [7] (81% in Ethiopia [8] and 51% in Guinea [9]), and a large share of postpartum depression remains undetected and untreated as a result. In addition, the majority of women who seek postnatal care in health facilities are underdiagnosed for postpartum depression due to poor availability of screening and treatment services in primary care settings in the region [10, 11].

A systematic review of studies from Ethiopia showed that 23% of postpartum women experience postpartum depression [12]. In Guinea, research on postpartum depression is very limited. However, stressors such as the Ebola crisis, political and economic instability, and the resulting weak health system performance in the country, are likely to contribute to a high prevalence of postpartum depression [13].

Given the increasing reports of postpartum depression among women who have been mistreated (disrespected and abused)during childbirth in health facilities [14–16], it is imperative to examine the role that mistreatment plays in the dynamics of postpartum depression, independently or with other known risk factors for postpartum depression. In September 2021, a high-level technical consultation led by the World Health Organization (WHO), the United Nations Population Fund (UNFPA), and the United States Agency for International Development (USAID) launched a call for evidence on the relationship between the mistreatment of women during facility-based childbirth and maternal mental health outcomes [17].

Mistreatment during childbirth could manifest in several ways, including verbal abuse, physical abuse, sexual abuse, stigma and discrimination, failure to meet professional standards of care, poor relationship between women and service providers, and health system conditions and constraints that can lead to poor quality of care and failure to meet women’s preferences [18]. Evidence from Ethiopia, Guinea, and other low- and middle-income countries shows a worryingly high prevalence (28%—83%) of mistreatment of women [19–21]. Mistreatment of women during childbirth not only undermines their right to a positive childbirth experience, but it also deters them from seeking future maternal and child health care, including care for postpartum depression [20]. However, there is a critical evidence gap on whether mistreatment of women during facility-based childbirth is associated with postpartum depression [17], which is particularly important in sub-Saharan Africa, where the use of antenatal care and facility-based childbirth is increasing dramatically [8, 9]. This study aims to estimate the independent association between the mistreatment of women during facility-based childbirth and postpartum depression and to explore health systems capacity to integrate respectful maternity care and maternal mental health services in urban settings in Ethiopia and Guinea.

## Methods

### Study setting

During recent decades, maternal health outcomes improved remarkably in Ethiopia primarily due to the significant progress in the accessibility of maternal health services [22]. However, suboptimal quality and inequity in the distribution of maternal health services between the urban–rural, and rich-poor remain a challenge, despite some improvements [22]. Ethiopia had a maternal mortality ratio of 267/100,000 live births in 2020 [23], indicating a significant gap to achieve the SDG target of having no country with a MMR of more than 140/100,000 live births by 2030. Ethiopia has a three-tiered decentralised health system. The country launched its first mental health strategy in 2012 [24] which was later followed by a five-year (2020—2025) national mental health strategic plan in 2020 [25]. The strategic plan aspires to 1) integrate maternal mental health into general health care including maternal, sexual, reproductive, and child health services, and 2) include maternal mental health as a key strategic pillar in the national maternal and child health strategy [25]. Addis Ababa, the capital of Ethiopia, had an estimated total population of more than 3.7 million in 2021 [26]. The city is administratively divided into 11 sub-cities. In 2021, there were 115 functioning public health facilities (13 hospitals and 102 health centres) and 21,497 health professionals registered in the city [26].

Guinea, a country with a pyramidal health system, is one of the countries with the worst maternal and newborn health indicators globally. Guinea is characterised by a fragile post-conflict context and the Ebola crisis that significantly affected its health system’s performance. Guinea had a maternal mortality ratio of 553/100,000 live births in 2020. Guinea launched its first mental health policy and national plan in 1995, later revised in 2000 and 2013 [27]. The plan aspires to integrate mental health services into primary health care and to drive the decentralisation of mental health services from hospitals to low-level facilities which are closer to the community [27]. However, contextual conditions and crises such as the Ebola virus disease outbreak between 2014 and 2015 have negatively affected the implementation of the plan [27]. Conakry, the capital of Guinea, had an estimated total population of more than 2 million in 2022. The city is administratively divided into six municipal communes. In 2022, there were 55 functioning public health facilities (10 hospitals and 45 health centres) in the city.

### Study design

The study uses a sequential mixed methods design, which will gather data from a longitudinal survey of women, desk review, and in-depth interviews with key stakeholders in the health system and women who recovered from postpartum depression. As indicated in Table 1, the project starts with a prospective longitudinal survey of women to measure perinatal (prenatal and postpartum) depression, estimate the proportion of postpartum depression that occurred after childbirth, and assess the independent association between mistreatment and postpartum depression (Study 1). Next, it focuses on exploring the integration of respectful maternity care and maternal mental health in Ethiopian and Guinean maternal health care systems (Study 2).

**Table 1:**
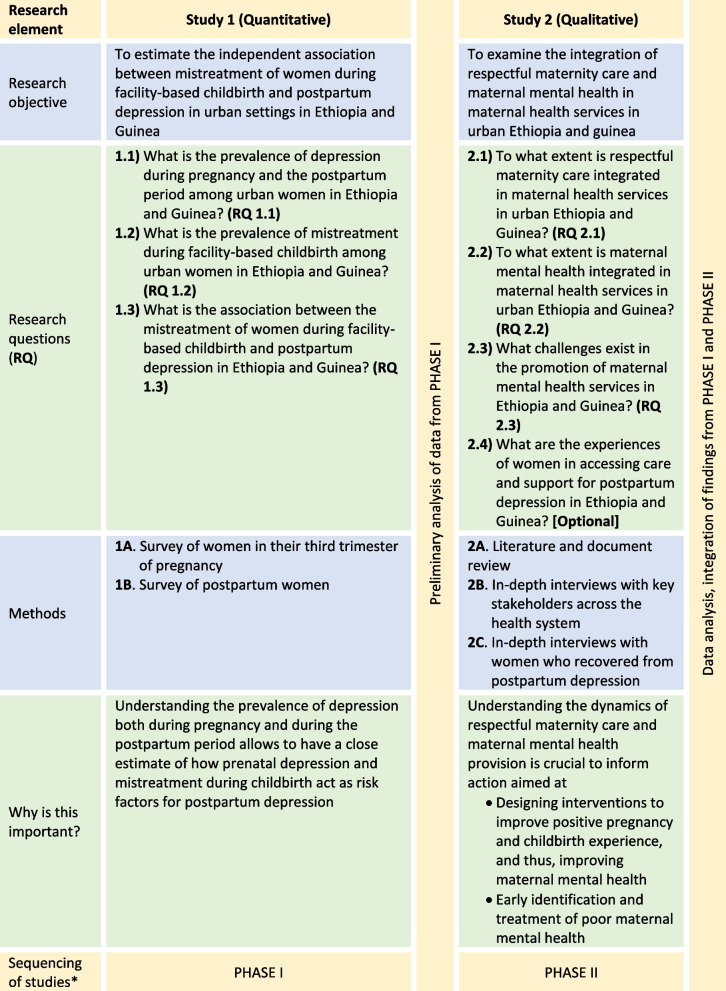
Summary of the research objectives, questions, methods, relevance and sequence across studies

We will apply a gendered lens during the design, analysis, and interpretation of findings of the project to have a better understanding of gender intersectionality. We will use the following approaches: including questions on gender issues as applied to mistreatment and maternal depression (Study 1); disaggregating data by gender (Study 2); examining power relations in the maternal health care system and beyond (Study 2), and using gender frameworks during the analysis of qualitative data (Study 1 & 2).

### Study 1

**Fig. 1:**
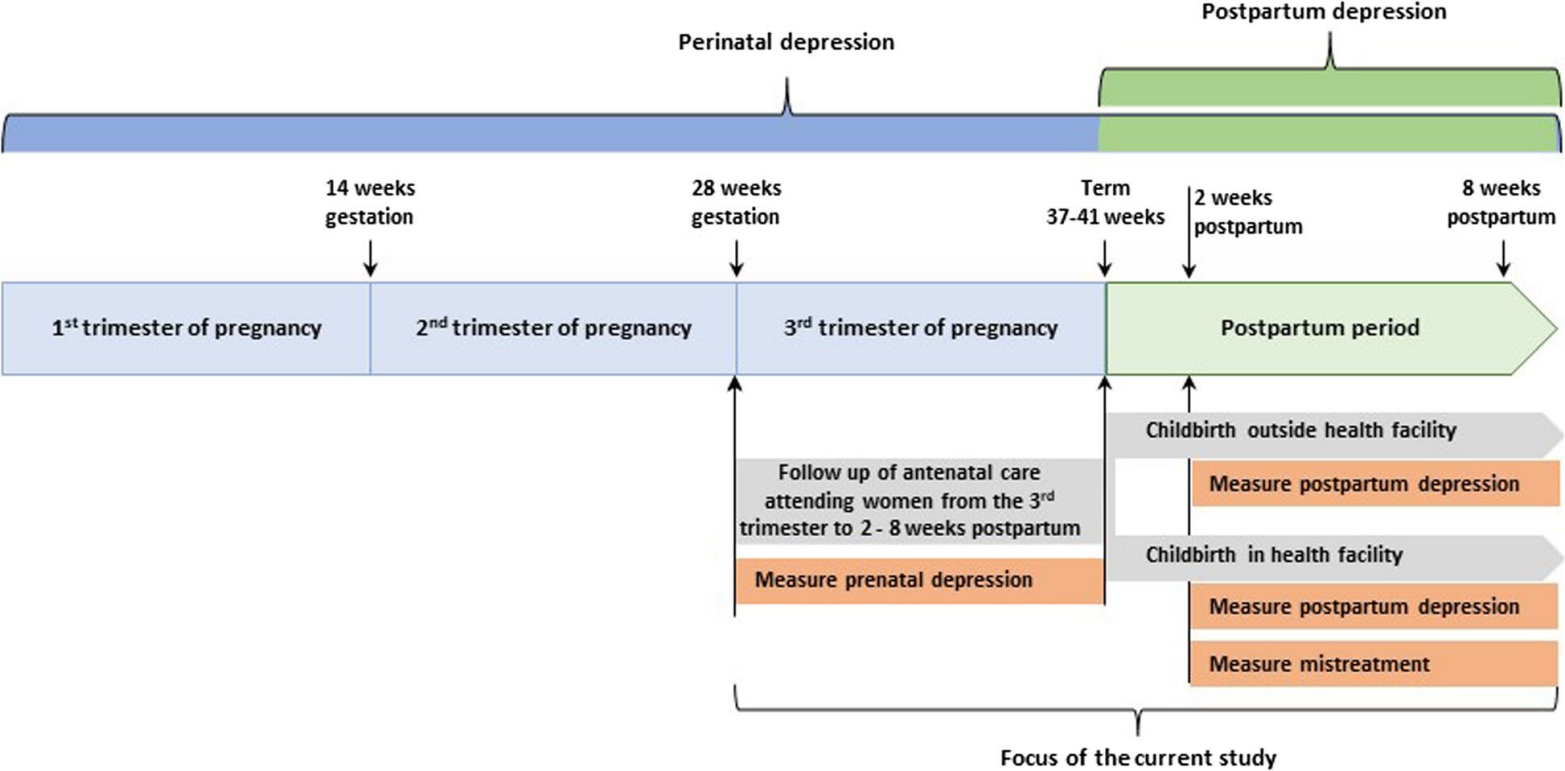
Focus of study 1

We will conduct a prospective longitudinal survey of pregnant women seeking antenatal care in health facilities in Addis Ababa (Ethiopia) and Conakry (Guinea). Women in the third trimester of pregnancy using antenatal care will be recruited and prospectively followed until two to eight weeks postpartum regardless of their mode or place of childbirth (Fig. 1). Almost all women in the two cities attend antenatal care in the third trimester of pregnancy and more than 89% give birth in health facilities (97% in Addis Ababa and 80.9% in Conakry) [8, 9]. Women will be surveyed two times—the first during the third trimester of pregnancy and the second during the postpartum period. The first survey will be facility-based and enrol women in the third trimester of pregnancy to measure the prevalence of prenatal depression (RQ 1.1). The second will be a community-based survey that follows up postpartum women who participated in the first round survey (two to eight weeks postpartum) to measure the prevalence of postpartum depression (RQ 1.1) and experience of mistreatment during facility-based childbirth (for those who gave birth in health facilities) (RQ 1.2). Accordingly, we will compare women’s depression scores before and after childbirth and estimate the independent association between the experience of mistreatment during facility-based childbirth and postpartum depression among those who gave birth in health facilities (RQ 1.3).

### Study 2

Study 2 will be conducted in two methodologically sequenced parts. We will first conduct a document review to understand the macro- and meso-level focus given to respectful maternity care and maternal mental health in maternal health services in Ethiopia and Guinea (RQ 2.1 & 2.2). The purpose here is to 1) understand how respectful maternity care and maternal mental health are organised at service delivery level and integrated into national policies and guidelines, and 2) ensure rigour of the qualitative data collection tool and interpretation of findings for RQ 2.3 [28]. Second, we will conduct in-depth interviews with a set of diverse stakeholders in the health system to explore the integration of respectful maternity care and maternal mental health in maternal health services provision (RQ 2.1 & 2.2) and challenges to the provision of respectful maternity care and maternal mental health services in urban settings in Ethiopia and Guinea (RQ 2.3). Depending on the availability of women following a clinically confirmed postpartum depression by a health expert (from those women who were included in the women’s survey), we will also conduct in-depth interviews with women to explore their experiences of care and support for postpartum depression they received from both the health system and other sources (RQ 2.4).

### Population and sample size

#### Study 1

Pregnant women attending antenatal care in health facilities in Addis Ababa and Conakry and who have lived in the cities for at least six consecutive months before the survey period will be recruited (1A – Table 1). Women in their third trimester of pregnancy (28 – 41 weeks gestation) will be informed about the purpose of the study at the end of their antenatal care consultations. Women who agree to participate in the study will be enrolled in the survey and provided with additional information regarding the second round survey which will take place at their home or their preferred place two to eight weeks postpartum (1B – Table 1).

A total of 842 women will be recruited into the study during the first round (434 in Addis Ababa and 408 in Conakry). This sample size was calculated using G*Power 3.1.9.7 software using the menu for estimating sample size for the difference between two dependent means (matched pairs) with the following assumptions: effect size of 0.2; 0.8 power; 0.05 level of significance; a design effect of 1.5; and a 15% chance of non-response or dropout (refusal or moving away from the study area). Additionally, the sample size was inflated to adjust for proportion of pregnant women who give birth outside of health facilities in urban settings in Ethiopia (21%) and Guinea (16%). To select women from Addis Ababa and Conakry, we will use a stratified cluster sampling method to achieve a representative sample of women who use facility-based antenatal care. The list of all eligible facilities will be obtained from Addis Ababa Regional Health Bureau and the Regional Department of Health in Conakry, and selection of facilities and women will be made as per the steps indicated in Table 2.

**Table 2:** Sampling procedures – study 1

Steps	Addis Ababa	Conakry
1	Based on the design effect (1.5) applied in the sample size calculation and the total sample size (434), it is methodologically sound to recruit 20 participants per cluster (health facility). Accordingly, 22 health facilities will be selected	Based on the design effect (1.5) applied in the sample size calculation and the total sample size (408), it is methodologically sound to recruit 20 participants per cluster (health facility). Accordingly, 20 health facilities will be selected
2	We stratify facilities by ownership (public and private). We regard non-governmental health facilities as private facilities as the proportion of women who give birth in those settings in Addis Ababa is very low	We stratify facilities by ownership (public, private, and faith-based)
3	We allocate the total sample size to the two sectors (public and private) based on the proportion of women who give birth in those settings (74% in public and 26% in private facilities). Accordingly, 330 and 114 women will be surveyed from public and private facilities, respectively. As per the cluster size in Step 1, 16 public and 6 private facilities will be selected	We allocate the total sample size to the three sectors (public, private, and faith-based) based on the proportion of women who give birth in those settings (75% in public, 20% in private and 2% in faith-based facilities). Accordingly, 306, 82, and 20 women will be surveyed from public, private, and faith-based facilities, respectively. As per the cluster size in Step 1, 15 public, 4 private, and 1 faith-based facilities will be selected
4	We allocate the 16 public health facilities to hospitals and health centres based on the distribution of births in public health facility-based births in the city – 48% in hospitals and 52% in health centres. Accordingly, 7 hospitals and 9 health centres will be randomly selected. Selection of the public hospitals and public health centres will again be made proportionally based on the number of childbirths at individual facilities in the same category (hospital or health centre) provided in the last quarter of 2022 (this information is available from Addis Ababa Regional Health Bureau)	We allocate the 15 public health facilities to hospitals, health centres, and health posts based on the distribution of births in public health facility-based births in the city – 34% in hospitals, 63% in health centres, and 3% in health posts; given the small proportion of births in health posts, we will merge these with health centres. Accordingly, 5 hospitals and 10 health centres will be randomly selected. Selection of the public hospitals and public health centres will again be made proportionally based on the number of childbirths at individual facilities in the same category (hospital or health centre) provided in the last quarter of 2022 (this information is available from Conakry Regional Department of Health)
5	We select the 6 private hospitals based on probability proportionate to size using the number of childbirths at individual private hospitals provided in the last quarter of 2022	We select the 4 private facilities based on probability proportionate to size using the number of childbirths at individual private facilities provided in the last quarter of 2022. Similarly, one faith-based facility will be selected based on probability proportionate to size using the number of childbirths at individual faith-based facilities provided in the last quarter of 2022
6	Eligible women accessing antenatal care in sampled health facilities in their third trimester of pregnancy will be consecutively invited to participate in the survey until the required sample (*n* = 20) from each selected facility is achieved	Eligible women accessing antenatal care in sampled health facilities in their third trimester of pregnancy will be consecutively invited to participate in the survey until the required sample (*n* = 20) from each selected facility is achieved

### *Study 2*

This component involves in-depth interviews with key-informants and women. Individual in-depth interviews with key-informants will involve frontline health workers who provide antenatal, intrapartum, and postnatal care in the selected health facilities in the two cities and maternal health program officers across the various levels of the health systems in the cities and at national level. The in-depth interviews with program officers will include heads of maternal health unit of health facilities, heads of facilities, and maternal health program coordinators at the sub-city, city, and national levels. We aim to conduct 40–50 in-depth interviews with key-informants (20–25 in each city) as this is likely to lead to saturation.

Depending on the availability of eligible participants, we will conduct 30–50 in-depth interviews with a sample of women (15–25 in each city) who have fully recovered from postpartum depression which has been clinically confirmed by health experts. These participants will be identified from the sample of women followed prospectively in Study 1, and the qualitative sampling will include considerations of capturing diversity in women’s profile such as socio-economic status, residence area, education, age, parity, mode of childbirth, and place of childbirth. This will be conducted at least six weeks after completion of the second round survey.

### Data collection

#### Study 1

Surveys will be conducted using interviewer-administered tablet-based questionnaires which will include tools to measure prenatal depression and mistreatment during antenatal care in the first round (Additional file 1) and postpartum depression and mistreatment during facility-based childbirth in the second round (Additional file 2). Prenatal and postnatal depression will be measured using the Edinburgh Postnatal Depression Scale (EPDS) [29] validated for use in Ethiopia [30] and in several West-African settings [31, 32]. Mistreatment during facility-based maternity care will be measured using a tool validated in sub-Saharan Africa [20] for use during pregnancy and in the postpartum period and adapted for use and tested in Ethiopia and Guinea. Additionally, data on risk factors for postpartum depression (socioeconomic status, stress, social support, marital satisfaction, spousal violence, history of depression) and obstetric characteristics (place of childbirth, mode of childbirth, obstetric complications) will be collected [5]. Social support will be measured using the *Maternity Social Support Scale* which involves six items with five-point Likert scales each [33]. Spousal violence in the 12 months preceding the survey date (first round survey) and since recent birth (second round survey) will be measured using a standard tool prepared by the DHS program and was part of the standard DHSs conducted in Ethiopia and Guinea [8, 9]. The questionnaires will be translated to (if not available already) and administered using Amharic in Addis Ababa and in Susu, Pular or Maninka in Conakry. Data will be collected digitally by female health professionals (nurses, midwives, or medical doctors) not employed in the study’s recruitment sites using the KoBoToolbox tool. There will also be two experienced field supervisors (one in each city) responsible for coordinating the overall field work. Three days’ training will be provided for the data collection team by the research team. The training will cover explanation of the study purpose, informed consent seeking, trust building, skills of interviewing, dealing with sensitive topics such as participants’ experience of spousal violence, distress management, and one-by-one explanation of contents of the questionnaire. Data collectors will practice the interview on each other before pre-testing the tool in selected health facilities in Addis Ababa and Conakry; each data collector will interview two women during the piloting phase under supervision, and receive feedback.

During the first round of survey in sampled health facilities, eligible women who have completed receipt of their routine antenatal care will be asked for their consent to participate in a survey which takes 15–25 min. If they agree, the first round of the survey will be administered immediately and their willingness to provide their contact address, including a phone number, will be noted. An Excel sheet that includes women’s gestational age at the time of the first survey, expected due date, and contact address will be prepared and used as a means to contact women during the postpartum period (2–8 weeks) when the second round survey will be administered at their homes or their preferred place. Completed questionnaires from women’s surveys will be uploaded to the online data collection platform by the data collectors – uploaded questionnaires will then be checked by the field supervisor and approved and protected by the principal investigator (PI) on a daily basis. Before starting the second round, refresher training will be provided to the data collectors. During this round of training, more focus will be given to skills of trust building and dealing with sensitive topics to build trust with participants—women may not be comfortable to speak about their negative experiences during childbirth in health facilities.

#### Study 2

A desk review of strategies, guidelines, policy briefs, training manuals, and other national-level thematic documents on maternal mental health and respectful maternity care in Ethiopia and Guinea will be conducted (RQ 2.1 & 2.2 – Table 1). In-depth interviews with key-informants will be conducted using a semi-structured question guide prepared using the recent WHO *Guide for integration of perinatal mental health in maternal and child health services* [34] (Additional file 3A and B); the guide will be further enriched after a review of recent global developments in maternal mental health provision in low-income settings (RQ 2.3). Additionally, the guide will be contextualised for Ethiopia and Guinea using the outputs of the document review (2A – Table 1) and preliminary analysis of data from Study 1. Cognisant of the potential disruptions in the countries’ health systems because of the COVID-19 pandemic, questions regarding how the pandemic affected provision of care will be included. The final question guides will be translated to Amharic (Ethiopia) and French (Guinea) which will be pre-tested and used to facilitate the interviews. Local researchers will facilitate the key-informant interviews with the PI.

In-depth interviews with women will be guided by a semi-structured guide (Additional file 4) which will be further enriched based on the findings of the women’s survey and review of the literature (RQ 2.4). In-depth interviews with women will be conducted by expert qualitative researchers who will be trained to provide psychosocial support where necessary.

### Data analysis

#### Study 1

Data will be analysed using StataSE v.17 (StataCorp, College Station, Texas, United States ). Women’s responses to the EPDS questions will be used to generate depression scores and identify those who have prenatal and postpartum depression. Depression scores before and after childbirth will be compared using three structural equation models.

i. Model 1—women who gave birth in health facilities and who did not have prenatal depression: this is a novel approach to account for prenatal depression and produce a robust estimate of the association between mistreatment and postpartum depression.
ii. Model 2—all women regardless of their prenatal depression scores and place of childbirth: this allows to compare the difference between prenatal and postpartum depression scores adjusting for women’s place of childbirth.
iii. Model 3—women who gave birth in health facilities regardless of their prenatal depression scores: this allows to have an estimate of the added scores of (severity) of postnatal depression due to mistreatment.

Multilevel mixed-effects Poisson regression will be used for modelling the depression scores before and after childbirth and estimate the independent effect of mistreatment. The levels will be facility ownership (public vs private), and facility types within these sectors (hospitals and health centres). All measured risk factors for postpartum depression (poor social support, marital dissatisfaction, spousal violence, pregnancy and/or childbirth complications, etc.), and experience of prenatal depression and mistreatment among participating women will be set as fixed effects, whereas the type of facility where women gave birth will be set as a random effect. Additionally, logistic regression will be used to estimate the adjusted odds of postpartum depression among women who were mistreated during childbirth in health facilities and who were not. Both regressions will be made by controlling for potential confounders (the various determinants of postpartum depression that have already been reported earlier in the Background) [5, 12].

#### Study 2

Identified documents on respectful maternity care will be reviewed using a framework for integrating respectful maternity care at the various levels of health systems [35] (RQ 2.1). Documents on maternal mental health will be reviewed using a framework developed by The Partnership for Maternal, Newborn and Child Health [36] to identify immediate- and long-term actions taken/designed to integrate maternal mental health at operational and policy levels in Ethiopia and Guinea (RQ 2.2). In-depth interviews with key stakeholders will be analysed using a hybrid thematic analysis (data- and theory-driven) approach assisted by NVivo software (QSR International, Version 1.4.1). The theory-driven aspect will be driven by the application of the complexity framework [37] (RQ 2.3). We will also apply a gender dimension during the analysis to explore how gendered power relations affect the provision of maternal mental health care and how that affects service providers [38]. A hybrid thematic analysis is a novel method of enhancing nuance, complexity and richness, and context in qualitative research that will allow understanding of the different contexts in Ethiopia and Guinea. Furthermore, the application of the complexity framework helps to understand the dynamics in maternal health systems in low-income settings where there are multiple and unpredictable interactions between system components.

### Data management

Data management procedures of our study comply to the Institute of Tropical Medicine’s research data management policy*.* Women’s surveys will not collect women’s personal identifiers. However, there will be a separate file (tracking sheet) to document participants’ names, phone numbers, and codes, which will be used to contact women for the second round survey. Completed tablet-based questionnaires from women’s surveys will be uploaded to the online data collection server (KoboToolbox) by the data collectors – uploaded questionnaires will then be checked by the field supervisor and approved and protected by the PI on a daily basis. Each participant’s online questionnaire will be allocated an alphanumeric code. We will use a separate password protected file which will have participants’ names, phone numbers, and codes. This form will only be accessed by the field supervisor to link the women’s responses to the first round survey with that of the second round and to easily identify women for the second round survey in the community. This file will be discarded after completion of the second round survey and will not be used on any subsequent documentation related to the study so that the participants name is not recorded anywhere else. The same pseudo anonymisation process will apply for respondents participating in the key-informant interviews; personal identifiers will not be collected during the in-depth interviews. Transcribed data will also be password encrypted. Upon satisfactory completion of transcription, audio recordings and short notes of qualitative interviews will be destroyed.

Electronic questionnaires and digital audio recordings of the in-depth interviews will be stored in a password-protected institutional server at the Institute of Tropical Medicine (ITM) on the same day the interviews will be made or as soon as access to internet connectivity is guaranteed. No personal identifiers will be stored on the electronic server. Raw data will only be shared with the project collaborators and will not be shared to an external party/person. Signed informed consent sheets will be stored in a locked cabinet. Electronic survey data, qualitative transcripts, and interview notes will finally be discarded five years after the last publication arising from this study.

## Discussion

Our study’s methodology is novel as it uses a longitudinal design to assess maternal depression during the third trimester of pregnancy and the postpartum period. This allows excluding maternal depression that might have existed before childbirth as a risk factor for postpartum depression, and makes possible a robust estimation of the independent association between mistreatment of women during childbirth and postpartum depression. The Poisson regression is intended to capture the worsening of depression scores due to mistreatment during childbirth among women who already had prenatal depression which cannot be captured by logistic regression. Poisson regression also allows examining whether the postpartum depression scores of women are significantly different based on women’s experiences of the various components of mistreatment, such as verbal abuse, physical abuse, non-consented care, etc. Additionally, it captures increased depression scores (antepartum vs postpartum), due to mistreatment in childbirth, among women who had prenatal depression. The inclusion of two different settings in our study (Addis Ababa and Conakry) will also enable us to apply a comparative health systems lens to explore the dynamics of respectful maternity care and maternal mental health services within the broader health systems of the two countries (Ethiopia and Guinea).

In light of the dearth of evidence on the prevalence of depression among pregnant and postpartum women in Ethiopia and Guinea, the study will fill the evidence gap in revealing the extent of the burden of prenatal and postpartum depression and its complex determinants. The findings of this study will provide policy guidance and evidence to develop interventions to 1) identify and mitigate the mistreatment of women during-facility based childbirth and thus reduce both mistreatment and postpartum depression (primary prevention), and 2) improve early detection and referral of women with perinatal depression (depression during pregnancy or the postpartum period) for adequate mental health care and support (secondary prevention and care). The findings will also be useful to point out actions that need to be taken to promote respectful maternity care and maternal mental health services through better integration. In the broader context, improving the childbirth experiences of women and thus their mental health enormously contributes to the achievement of the maternal health and mental health targets of the Sustainable Development Goals (SDGs).

## Supplementary Information

Additional file 1: Questionnaire for women’s survey during pregnancy.

Additional file 2: Questionnaire for women’s survey during the postpartum period.

Additional file 3A and 3B: Interview guides for in-depth interviews with key-informants.

Additional file 4: Interview guides for in-depth interviews with women.

## Data Availability

No datasets were generated or analysed during the current study.

## Abbreviations

COVID-19: Coronavirus Disease
EPDS: Edinburgh Postnatal Depression Scale
FWO: Research Foundation-Flanders
ITM: Institute of Tropical Medicine, Antwerp
PI: Principal Investigator
RQ: Research Question
SDGs: Sustainable Development Goals
UNFPA: United Nations Population Fund
USAID: United States Agency for International Development
WHO: World Health Organization
