# Supplementary material for: Is MIStreatment of women during facility-based childbirth an independent risk factor for POstpartum Depression in Ethiopia and Guinea? A mixed methods prospective study protocol—MISPOD study
Source: Reprod Health. 2024 Sep 4;21:129. doi: 10.1186/s12978-024-01850-w (PMC11375852; doi:10.1186/s12978-024-01850-w)
Supplement: Supplementary file 1 — Additional file 1: Questionnaire for women’s survey during pregnancy. [file 12978_2024_1850_MOESM1_ESM.docx]

**Additional file 1: Questionnaire for women’s survey during pregnancy**

Name of data collector፡____________________________________

**Screening questions**

Have you been living in Addis Ababa/Conakry over the last six months?

Yes  No  *If “No”, thank the participant and stop the survey.*

What is your current gestational age (in weeks)? _______weeks. *If “<28 weeks”, thank the participant and stop the survey*

Health Facility: ________________________

Four-digit questionnaire code: ___________

Date of data collection: ______________

Time of data collection (start): ______________

**Section I: Socio-demographic and household characteristics**

| **S.No** | **Questions** | **Possible responses** | **Remark/skip** |
| --- | --- | --- | --- |
|  | In which sub-city/commune do you live? | ________________ |  |
|  | How long have you lived in Addis Ababa/Conakry?  *Note: Round off using 6 months as a cut-off point. Example:1 year and 6 months should be written as 2 years* | ––––––––years |  |
|  | What is your current age/how old are you today (in completed years)? | 1. ­­­­­––––––––––––––– years 2. Don’t know/refused to answer |  |
|  | How old were you at your first pregnancy (in completed years)? | 1. ­­­­­––––––––––––––– years 2. Don’t know/refused to answer |  |
|  | What is your current marital status? | 1. Single 2. Married 3. Cohabiting 4. Separated 5. Divorced 6. Widowed 7. Don’t know / refused to answer |  |
|  | What is your highest educational status? | 1. No education 2. Primary - not completed grade 8 3. Completed grade 8 4. Secondary - not completed grade 12 5. Completed grade 12 6. More than secondary 7. Don’t know / refused to answer |  |
|  | What is your current occupation? | 1. Housewife 2. Farmer 3. Private Employee 4. Government Employee 5. Private business 6. Others: specify.................... 7. Don’t know / refused to answer |  |
|  | How many alive child/children do you have? | 1. _______________child/children 2. Don’t know / refused to answer |  |
|  | How many people (including adults and children, and including you) live in your household? | 1. _______________persons 2. Don’t know / refused to answer |  |
|  | Do you have regular household monthly cash income? | 1. Yes 2. No 3. Don’t know / refused to answer | **If 2/3 🡪 114** |
|  | What is your household’s monthly income (in Birr)? | 1. ––––––––––––––– Eth. Birr 2. Don’t know / refused to answer |  |
|  | In the past four weeks, how often have you worried that you cannot feed your family? | 1. Not at all 2. Hardly ever 3. Sometimes 4. Very often 5. Don’t know / refused to answer |  |

**Section II: Obstetric and previous service utilisation characteristics**

| **S.No** | **Questions** | **Possible responses** | **Remark/skip** |
| --- | --- | --- | --- |
|  | How many times have you given birth before? | ___________________ times | **If 0 🡪 204** |
|  | Where did you give birth your last (previous) child? | 1. At health facility 2. At home 3. At traditional birth attendant’s home 4. On the way to health facility 5. Others: specify.................... 6. Don’t know / refused to answer |  |
|  | How many times have you given birth in a health facility? | 1. ___________________ times 2. Don’t know / refused to answer |  |
|  | Was your current pregnancy planned at the time you got pregnant? | 1. Yes, I wanted a child now 2. No, I wanted a child later 3. No, I did not want a child at all |  |
|  | Are you pregnant with one baby or twins? | 1. Singleton 2. Multiple (two or more) 3. I don’t know |  |
|  | How many antenatal care visits did you have during your current pregnancy, including your visit today? | 1. ________ visit/s 2. Don’t know / refused to answer |  |
|  | Have you had any complications during your current pregnancy? | 1. Yes, specify ____________ 2. No 3. Don’t know / refused to answer |  |

**Section III: Social support and marital satisfaction**

Read this for the woman: “the following statements are related to social support you receive from your husband/partner/families /relatives/friends. I will read the statements one by one; you have five options to choose from”.

| **S.No** | **Questions** | **Possible responses** | | | | | |
| --- | --- | --- | --- | --- | --- | --- | --- |
| ***For each of the following statements, please circle one response which shows how the woman feels about the support she has right now*** | | **Always** | **Most of the time** | **Some of the time** | **Rarely** | **Never** | **Don’t know or refused** |
|  | I have good friends who support me | 5 | 4 | 3 | 2 | 1 | 99 |
|  | My family is always there for me | 5 | 4 | 3 | 2 | 1 | 99 |
|  | My husband/partner helps me a lot  ***Note: skip if the woman is NOT married or cohabited*** | 5 | 4 | 3 | 2 | 1 | 99 |
|  | There is conflict with my husband/partner  ***Note: skip if the woman is NOT married or cohabited*** | 1 | 2 | 3 | 4 | 5 | 99 |
|  | I feel controlled by my husband/partner  ***Note: skip if the woman is NOT married or cohabited*** | 1 | 2 | 3 | 4 | 5 | 99 |
|  | I feel loved by my husband/partner  ***Note: skip if the woman is NOT married or cohabited*** | 5 | 4 | 3 | 2 | 1 | 99 |

**Section IV: Respectful antenatal care**

Read this for the woman: the following questions are related to your antenatal care experience in your current visit. I will read the questions one by one; you have three options to choose from: “Yes” or “No” or “NA (Not applicable)”. Dear data collector, mark only on the corresponding response

| **S.No** | **Questions** | **Possible responses** | | | **Remark/skip** |
| --- | --- | --- | --- | --- | --- |
| ***During your current antenatal check:*** | | **Yes** | **No** | **NA/refused** |  |
|  | Did the health workers use harsh or rude language? | 1 | 0 | 3 |  |
|  | Did the health workers make judgmental or accusatory comments about you? | 1 | 0 | 3 |  |
|  | Were you beaten, slapped, kicked, or pinched? | 1 | 0 | 3 |  |
|  | Were you gagged? | 1 | 0 | 3 |  |
|  | Were you physically restrained? | 1 | 0 | 3 |  |
|  | Did the health workers make threats of withholding treatment? | 1 | 0 | 3 |  |
|  | Did the health worker/s blame you for being pregnant or for any aspects of your pregnancy? | 1 | 0 | 3 |  |
|  | Did the health workers obtain your consent for all procedures, including physical examination? | 1 | 0 | 3 |  |
|  | Did the health workers keep information about you confidential? | 1 | 0 | 3 |  |
|  | Were you left in the antenatal care clinic for a prolonged period of time without attention? | 1 | 0 | 3 |  |
|  | Did the health workers conduct an abdominal examination for you? |  |  |  | **If 0/3 🡪414** |
|  | Did the health workers ask your permission before conducting an abdominal examination? | 1 | 0 | 3 |  |
|  | Did the health workers conduct abdominal examination without maintaining your privacy? | 1 | 0 | 3 |  |
|  | Did the health workers speak to you in a language you do not understand? | 1 | 0 | 3 |  |
|  | Did you want to have a companion in the antenatal clinic? | 1 | 0 | 3 | **If 0/3 🡪417** |
|  | Did the health workers allow you to have your companion present? | 1 | 0 | 3 |  |
|  | Did the health workers make you stay in the health facility against your will? | 1 | 0 | 3 |  |
|  | Did the health workers discriminate against you based on your religion /ethnicity/age/socioeconomic status/medical condition? | 1 | 0 | 3 |  |
|  | Do you think you have been mistreated (disrespected or abused) by a health worker during your antenatal visit today? | 1. Yes 2. No 3. Don’t know or refused to answer | | |  |

**Section V: Antepartum depression screening**

Read this for the woman: the following statements are related to your experience of mental health in the past 7 days. I will read the statements one by one; you have four options to choose from.

***Dear data collector, circle only on the corresponding response the woman made***

| **S.No** | **Questions** | **Possible responses** | **Remark** | |  |
| --- | --- | --- | --- | --- | --- |
| ***In the past 7 days*** | | | |  | |
|  | I have been able to laugh and see the funny side of things | 1. As much as I always could 2. Not quite so much now 3. Definitely not so much now 4. Not at all |  | |  |
|  | I have looked forward with enjoyment to things | 1. As much as I ever did 2. Rather less than I used to 3. Definitely less than I used to 4. Hardly at all |  | |  |
|  | I have blamed myself unnecessarily when things went wrong | 1. Yes, most of the time 2. Yes, some of the time 3. Not very often 4. No, never |  | |  |
|  | I have been anxious or worried for no good reason | 1. No, not at all 2. Hardly ever 3. Yes, sometimes 4. Yes, very often |  | |  |
|  | I have felt scared or panicky for no very good reason | 1. Yes, quite a lot 2. Yes, sometimes 3. No, not much 4. No, not at all |  | |  |
|  | Things have been getting on top of me | 1. Yes, most of the time I haven’t been able to cope at all 2. Yes, sometimes I haven’t been coping as well as usual 3. No, most of the time I have coped quite well 4. No, I have been coping as well as ever |  | |  |
|  | I have been so unhappy that I have had difficulty sleeping | 1. Yes, most of the time 2. Yes, sometimes 3. Not very often 4. No, not at all |  | |  |
|  | I have felt sad or miserable | 1. Yes, most of the time 2. Yes, sometimes 3. Not very often 4. No, not at all |  | |  |
|  | I have been so unhappy that I have been crying | 1. Yes, most of the time 2. Yes, quite often 3. Only occasionally 4. No, never |  | |  |
|  | The thought of harming myself has occurred to me | 1. Yes, quite often 2. Sometimes 3. Hardly ever 4. Never |  | |  |
|  | Did you have any history of depression before your current/recent pregnancy? | 1. Yes 2. No 3. I don’t know |  | |  |

**Section VI: Spousal violence**

***Screening question***

**How long have you been married or cohabited – only current union (months)? ______ months**

***Note: skip this section for women who were not married or cohabited in the 12 months preceding the survey***

Enumerator: ensure auditory privacy!

Read this for the woman: the following statements are related to your experience of violence by your partner/husband in the past 12 months. I will read the statements one by one; you have three options to choose from “Yes” or “No” or “I don’t know”.

| **S.No** | **Questions** | **Possible responses** | | | **Remark/skip** |
| --- | --- | --- | --- | --- | --- |
| ***In the last 12 months, did your last husband/partner ever:*** | | **Yes** | **No** | **I don’t know** |  |
|  | Push you, shake you, or throw something at you? | 1 | 0 | 3 |  |
|  | Slap you or twist your arm? | 1 | 0 | 3 |  |
|  | Punch you with his fist or with something that could hurt you? | 1 | 0 | 3 |  |
|  | Kick you or drag you? | 1 | 0 | 3 |  |
|  | Try to strangle you or burn you? | 1 | 0 | 3 |  |
|  | Threaten you with a knife, gun, or other type of weapon? | 1 | 0 | 3 |  |
|  | Attack you with a knife, gun, or other type of weapon? | 1 | 0 | 3 |  |
|  | Physically force you to have sexual intercourse with him even when you did not want to? | 1 | 0 | 3 |  |
|  | Force you to perform other sexual acts you did not want to? | 1 | 0 | 3 |  |
|  | Said or did something to humiliate you in front of others? | 1 | 0 | 3 |  |
|  | Threatened to hurt or harm you or someone you cared about? | 1 | 0 | 3 |  |
|  | Insulted you or made you feel bad about yourself? | 1 | 0 | 3 |  |

**Thank you for your participation!**

Time (end of survey): _____________
