# Supplementary material for: Is MIStreatment of women during facility-based childbirth an independent risk factor for POstpartum Depression in Ethiopia and Guinea? A mixed methods prospective study protocol—MISPOD study
Source: Reprod Health. 2024 Sep 4;21:129. doi: 10.1186/s12978-024-01850-w (PMC11375852; doi:10.1186/s12978-024-01850-w)
Supplement: Supplementary file 2 — Additional file 2: Questionnaire for women’s survey during the postpartum period. [file 12978_2024_1850_MOESM2_ESM.docx]

**Additional file 2: Questionnaire for women’s survey during the postpartum period**

Sub-city/commune (place of data collection): ________________________

Four-digit questionnaire code: ___________

Date of data collection: ______________

Time of data collection (start): ______________

Place of data collection: At woman’s house  Health facility  Other (specify) _____________

**Section I: Socio-demographic and household characteristics (update from round 1)**

**Can I confirm that some information we have about you is still correct?**

| **S.No** | **Questions** | **Possible responses** | **Remark/skip** |
| --- | --- | --- | --- |
| 101 | Current marital Status | 1. Single 2. Married 3. Cohabiting 4. Separated 5. Divorced 6. Widowed 7. Don’t know / refused to answer |  |
| 102 | In the past four weeks, how often have you worried that you cannot feed your family?  ***Please read the options*** | 1. Not at all 2. Hardly ever 3. Sometimes 4. Very often 5. Don’t know / refused to answer |  |

**Section II: Social support and marital satisfaction**

Read this for the woman: “the following statements are related to social support you receive from your husband/partner/families /relatives/friends. I will read the statements one by one; you have five options to choose from”.

| **S.No** | **Questions** | **Possible responses** | | | | | |
| --- | --- | --- | --- | --- | --- | --- | --- |
| ***For each of the following statements, please circle one response which shows how the woman feels about the support she has right now*** | | **Always** | **Most of the time** | **Some of the time** | **Rarely** | **Never** | **Don’t know or refused** |
|  | I have good friends who support me | 5 | 4 | 3 | 2 | 1 | 99 |
|  | My family is always there for me | 5 | 4 | 3 | 2 | 1 | 99 |
|  | My husband/partner helps me a lot  ***Note: skip if the woman is NOT married or cohabited*** | 5 | 4 | 3 | 2 | 1 | 99 |
|  | There is conflict with my husband/partner  ***Note: skip if the woman is NOT married or cohabited*** | 1 | 2 | 3 | 4 | 5 | 99 |
|  | I feel controlled by my husband/partner  ***Note: skip if the woman is NOT married or cohabited*** | 1 | 2 | 3 | 4 | 5 | 99 |
|  | I feel loved by my husband/partner  ***Note: skip if the woman is NOT married or cohabited*** | 5 | 4 | 3 | 2 | 1 | 99 |

**Section III: current childbirth related characteristics**

| **S.No** | **Questions** | **Possible responses** | | | | | **Remark/skip** |
| --- | --- | --- | --- | --- | --- | --- | --- |
|  | When did you give birth? | *___dd___mm___yy* | | | | |  |
|  | What was the outcome of the participant’s recent pregnancy?  *(do not ask this question; rather complete based on what you see or the participant’s responses to other questions/initial phone call)* | 1. Live birth 2. Live birth but infant death afterward, specify age at death _______days 3. Stillbirth | | | | |  |
|  | Did you have any complication during your recent pregnancy? | 1. Yes, specify ____________ 2. No 3. Don’t know / refused to answer | | | | |  |
|  | Did you have any complication during your childbirth? | 1. Yes, specify ______________ 2. No 3. Don’t know / refused to answer | | | | |  |
|  | Where did you give birth? | 1. At a health facility 2. On the way to health facility 3. At home and went to health facility thereafter 4. At home 5. At traditional birth attendant’s home/other home setting | | | | | **If 4/5 🡪 319** |
|  | Facility name | ____________________  __________________ | | | | |  |
|  | Do you know what kind of facility this is? | 1. Public health centre 2. Public general/referral hospital 3. Public specialised teaching hospital 4. Private hospital 5. Private higher clinic 6. Private not-for-profit 7. Don’t know | | | | |  |
|  | Were you referred to this facility from another facility or directly went to the facility for childbirth? | 1. Referred from another facility 2. Non-referred (direct visit) | | | | | **If 2 🡪 310** |
|  | How many health facilities did you visit as part of the referral process? | __________________ | | | | |  |
|  | When did you get admitted to the health facility you gave birth at or you visited after giving birth at home? | _______AM *(___dd___mm___yy)*  _______PM *(___dd___mm___yy)* | | | | |  |
|  | What time did you give birth? | _______AM *(___dd___mm___yy)*  _______PM *(___dd___mm___yy)* | | | | |  |
|  | What type of ward were you in? | 1. Private ward 2. Shared ward 3. Don’t know / refused to answer | | | | |  |
|  | What was the gender of the service provider who mainly assisted you during your labour and childbirth? | 1. Female 2. Male 3. Don’t know | | | | |  |
|  | What type of birth did you have? | 1. Vaginal birth 2. Assisted vaginal birth 3. Caesarean birth after labour trial 4. Caesarean birth without labour trial 5. Don’t know | | | | |  |
|  | Did you have any procedure for an assisted delivery? | 1. Yes 2. No 3. DK/refused | | | | | **If 2/3 🡪 317** |
|  | Which procedure did you receive? ***(Multiple responses possible)*** |  | | | **Yes** | **No** |  |
|  |  | Vacuum | | | 1 | 2 |  |
|  |  | Forceps | | | 1 | 2 |  |
|  |  | Episiotomy (not tear) | | | 1 | 2 |  |
|  | How long did you stay at the health facility? | 1. _____ number of hours 2. _____ number of days 3. _____ number of weeks 4. don’t know or refused to answer | | | | |  |
|  | Did you have a postnatal check by a health professional before you were discharged from the health facility after your childbirth?  ***NB: skip if the woman gave birth at home*** | 1. Yes 2. No | | | | |  |
|  | Did you have a postnatal check by a health professional after your childbirth?  *(NB: postnatal check after discharge if birth happened at a health facility)* | 1. Yes, at home 2. Yes, at a health facility 3. Yes, both at home and a health facility 4. No 5. Don’t know or refused to answer | | | | |  |
|  | Did you intend to breastfeed your baby? ***NB: skip if the woman’s response to Q302 is 2/3*** | 1. Yes 2. No | | | | | ***If 2/3 for Q302* 🡪 401** |
|  | Did you breastfeed yesterday sunrise to today sunrise? | 1. Yes 2. No 3. Don’t know or refused to answer | | | | |  |
|  | Did you have to stop breastfeeding due to circumstances beyond your control? | 1. Yes 2. No 3. Don’t know or refused to answer | | | | |  |
|  | How happy are you with your breastfeeding practice?  *Please read the options* | 1. Very happy 2. Happy 3. Neutral 4. Unhappy 5. Very unhappy 6. Don’t know or refused to answer | | | | |  |
|  | Do you have a card or another document where your newborn’s vaccinations are written down? | 1. Yes, card or another document seen 2. No, no card or other document seen | | | | | **If 2 🡪 327** |
|  | *Data collector: copy the following information from the vaccination card or document* |  | Yes | No | | |  |
|  |  | BCG |  |  | | |  |
|  |  | Polio-0 |  |  | | |  |
|  |  | Polio-1 |  |  | | |  |
|  |  | Penta-1 |  |  | | |  |
|  | I want to take the picture of the newborn’s vaccination card or document so that it will help me to counter check the information I filled in. Are you willing?  *Data collector: if the caregiver consented take the snapshot and upload the picture*. | 1. Yes 2. No | | | | |  |
|  | Has the newborn received a BCG vaccination against tuberculosis, that is, an injection in the arm or shoulder that usually causes a scar? | 1. Yes 2. No 3. I don’t know/refused to answer | | | | |  |
|  | Has the newborn received oral polio vaccine, that is, two drops in the mouth to prevent polio? | 1. Yes 2. No 3. I don’t know/refused to answer | | | | | **If 2/3 🡪 331** |
|  | Did the newborn receive the first oral polio vaccine in the first two weeks after birth or later? | 1. In the first two weeks 2. Later | | | | |  |
|  | How many times did the newborn receive the oral polio vaccine? | __________ times | | | | |  |
|  | Has the newborn ever received a pentavalent vaccination, that is, an injection usually given on the left upper thigh sometimes at the same time as polio drops? | 1. Yes 2. No 3. I don’t know/refused to answer | | | | |  |

**Section IV: Respectful maternity/childbirth care**

Read this for the woman: the following questions are related to your experience in your current childbirth. I will read the questions one by one; you have three options to choose from: “Yes” or “No” or “DK (I do not know)”. Dear data collector, select only one corresponding response.

***!! Skip this section if response to Q305 is 4/5***

| **S.No** | **Question** | **Possible responses** | | | **Remark/skip** |
| --- | --- | --- | --- | --- | --- |
| ***During this admission for childbirth…*** | | **Yes** | **No** | **DK** |  |
|  | Did the health workers use harsh or rude language? | 1 | 0 | 3 |  |
|  | Did the health workers make judgmental or accusatory comments about you? | 1 | 0 | 3 |  |
|  | Were you beaten, slapped, kicked, or pinched during childbirth? | 1 | 0 | 3 |  |
|  | Were you gagged during childbirth? | 1 | 0 | 3 |  |
|  | Were you physically restrained during childbirth? | 1 | 0 | 3 |  |
|  | Did the health workers make threats of withholding treatment? | 1 | 0 | 3 |  |
|  | Did the health workers blame you for any feature of your birth outcomes? | 1 | 0 | 3 |  |
|  | Did the health workers obtain your consent for all procedures? | 1 | 0 | 3 |  |
|  | Did the health workers keep information about you confidential? | 1 | 0 | 3 |  |
|  | Did you have any surgical procedure (episiotomy, caesarean section)? | 1 | 0 | 3 | **If 0/3 🡪 412** |
|  | Did the provider ask your permission before performing the surgical procedure? | 1 | 0 | 3 |  |
|  | Did the health workers always come following your call? | 1 | 0 | 3 |  |
|  | Were you ever left for a prolonged period of time without attention during your labour or postpartum care? | 1 | 0 | 3 |  |
|  | Was a health provider present for the actual birth of your baby? | 1 | 0 | 3 | **Skip if 2/3/4/5 for Q 305** |
|  | Did the health workers ever separate you from your baby without explaining the reason? | 1 | 0 | 3 |  |
|  | Did the health workers ask your permission before conducting a vaginal examination? | 1 | 0 | 3 |  |
|  | Did any provider conduct vaginal examination without maintaining your privacy? | 1 | 0 | 3 |  |
|  | Did the health workers speak to you in a language you do not understand? | 1 | 0 | 3 |  |
|  | Did the health workers give you periodic updates on your labor? | 1 | 0 | 3 | **Skip if 2/3/4/5 for Q 305** |
|  | Did you want to have a birth companion during your labour and childbirth in the labor ward? | 1 | 0 | 3 | **Skip if 2/3/4/5 for Q 305**  **If 0 🡪422** |
|  | Did the health workers allow you to have your birth companion present? | 1 | 0 | 3 |  |
|  | Did you want to move around during your labor? | 1 | 0 | 3 | **Skip if 2/3/4/5 for Q 305**  **If 0 🡪424** |
|  | Did the health workers allow you to move around during your labor? | 1 | 0 | 3 |  |
|  | Did you want to have food or fluids during your labor? | 1 | 0 | 3 | **Skip if 2/3/4/5 for Q 305**  **If 0 🡪426** |
|  | Did the health workers allow you to have food or fluids? | 1 | 0 | 3 |  |
|  | Did you have a preferred birthing position? | 1 | 0 | 3 | **Skip if 2/3/4/5 for Q 305**  **If 0 🡪428** |
|  | Did the health workers allow you to give birth in your preferred position? | 1 | 0 | 3 |  |
|  | Did you want to have a cultural practice in labor? | 1 | 0 | 3 | **Skip if 2/3/4/5 for Q 305**  **If 0 🡪430** |
|  | Did the health workers allow you this cultural practice in labor? | 1 | 0 | 3 |  |
|  | Did the health workers make you stay in the health facility against your will? | 1 | 0 | 3 |  |
|  | Did the health workers discriminate against you based on your religion /ethnicity/age/socioeconomic status/medical condition? | 1 | 0 | 3 |  |
|  | Do you think you have been mistreated by a health worker during your recent childbirth? | 1 | 0 | 3 |  |
|  | Have you been mistreated by other people outside the maternity care setting (for example guards, registration desk officers, other administrative and support staff, etc)? | 1 | 0 | 3 |  |

**Section V: Postpartum depression screening questions**

Read this for the woman: the following statements are related to your experience of mental health in the past 7 days. I will read the statements one by one; you have four options to choose from.

***Dear data collector, circle only on the corresponding response the woman made***

| **S.No** | **Questions** | **Possible responses** | **Remark** |
| --- | --- | --- | --- |
| ***In the past 7 days*** | | |  |
|  | I have been able to laugh and see the funny side of things | 1. As much as I always could 2. Not quite so much now 3. Definitely not so much now 4. Not at all |  |
|  | I have looked forward with enjoyment to things | 1. As much as I ever did 2. Rather less than I used to 3. Definitely less than I used to 4. Hardly at all |  |
|  | I have blamed myself unnecessarily when things went wrong | 1. Yes, most of the time 2. Yes, some of the time 3. Not very often 4. No, never |  |
|  | I have been anxious or worried for no good reason | 1. No, not at all 2. Hardly ever 3. Yes, sometimes 4. Yes, very often |  |
|  | I have felt scared or panicky for no very good reason | 1. Yes, quite a lot 2. Yes, sometimes 3. No, not much 4. No, not at all |  |
|  | Things have been getting on top of me | 1. Yes, most of the time I haven’t been able to cope at all 2. Yes, sometimes I haven’t been coping as well as usual 3. No, most of the time I have coped quite well 4. No, I have been coping as well as ever |  |
|  | I have been so unhappy that I have had difficulty sleeping | 1. Yes, most of the time 2. Yes, sometimes 3. Not very often 4. No, not at all |  |
|  | I have felt sad or miserable | 1. Yes, most of the time 2. Yes, sometimes 3. Not very often 4. No, not at all |  |
|  | I have been so unhappy that I have been crying | 1. Yes, most of the time 2. Yes, quite often 3. Only occasionally 4. No, never |  |
|  | The thought of harming myself has occurred to me | 1. Yes, quite often 2. Sometimes 3. Hardly ever 4. Never |  |

**Section VI: Spousal violence**

Enumerator: ensure auditory privacy!

***Note: only ask if the woman who was married/cohabiting since her recent birth***

Read this for the woman: the following statements are related to your experience of violence by your partner/husband since your recent birth. I will read the statements one by one; you have three options to choose from.

| **S.No** | **Questions** | **Possible responses** | | | **Remark/skip** |
| --- | --- | --- | --- | --- | --- |
| ***Since your recent birth, did your last husband/partner ever:*** | | **Yes** | **No** | **I don’t know** |  |
|  | Push you, shake you, or throw something at you? | 1 | 0 | 3 |  |
|  | Slap you or twist your arm? | 1 | 0 | 3 |  |
|  | Punch you with his fist or with something that could hurt you? | 1 | 0 | 3 |  |
|  | Kick you or drag you? | 1 | 0 | 3 |  |
|  | Try to strangle you or burn | 1 | 0 | 3 |  |
|  | Threaten you with a knife, gun, or other type of weapon? | 1 | 0 | 3 |  |
|  | Attack you with a knife, gun, or other type of weapon? | 1 | 0 | 3 |  |
|  | Physically force you to have sexual intercourse with him even when you did not want to? | 1 | 0 | 3 |  |
|  | Force you to perform other sexual acts you did not want to? | 1 | 0 | 3 |  |
|  | Said or did something to humiliate you in front of others? | 1 | 0 | 3 |  |
|  | Threatened to hurt or harm you or someone you cared about? | 1 | 0 | 3 |  |
|  | Insulted you or made you feel bad about yourself? | 1 | 0 | 3 |  |

**Thank you for your participation!**

Time (end of survey): _____________
