# Supplementary material for: Is MIStreatment of women during facility-based childbirth an independent risk factor for POstpartum Depression in Ethiopia and Guinea? A mixed methods prospective study protocol—MISPOD study
Source: Reprod Health. 2024 Sep 4;21:129. doi: 10.1186/s12978-024-01850-w (PMC11375852; doi:10.1186/s12978-024-01850-w)
Supplement: Supplementary file 3 — Additional file 3A and 3B: Interview guides for in-depth interviews with key-informants. [file 12978_2024_1850_MOESM3_ESM.docx]

**Additional file 3A: In-depth interview guide for interviews at higher health-system-levels**

***Background characteristics of key-informant***

| *Organisation* |  |
| --- | --- |
| *Position* |  |
| *Current profession* |  |
| *Previous role in health facilities (Y/N)* |  |
| *Service year in current position* |  |
| *Service year in the health system* |  |
| *Interview date and time* | *__dd/__mm__yy From __:__ am/pm* ***to*** *__:__am/pm* |

**Interview guide**

1. In your opinion what are the characteristics/domains of high-quality care for women at the time of labour and childbirth?
   - *Explore: infrastructure, human resource, client-provider relationships, mental health needs of women*
2. Would you please describe the forms of mistreatment (disrespect and abuse) women experience during childbirth in health facilities?
   - *Provide clear explanation of what mistreatment is when saturation of ideas is reached for this question*
3. It is reported that women are sometimes disrespected and even abused at the time of labour and childbirth in health facilities in Addis Ababa/Ethiopia (Conakry/Guinea). In your opinion, what factors contribute to this problem?
   - *Explore: providers’ knowledge and attitude, staff shortage, staff motivation, team spirit, infrastructure, client load, etc*
4. Would you please describe respectful maternity care?
   - *Provide clear explanation of what respectful maternity care is when saturation of ideas is reached for this question*
5. What do you think is the status of respectful maternity care in health facilities in Addis Ababa/Ethiopia (Conakry/Guinea)?
   - *Explore: privacy, confidentiality, client-provider interaction, complaints on non-respectful care, etc*
6. How do the health facilities/sub-cities/regions in your administrative scope actively promote respectful maternity care? If they don’t, why not?
   - *Explore: ongoing initiatives, guidelines, IEC/BCC activities, observe maternity settings*
7. What are the challenges to promote respectful maternity care in your administrative scope?
   - *Explore: providers knowledge and attitude, staff availability, staff motivation, team spirit, infrastructure, client load, promoting providers’ right, etc*
8. What measures are being taken to ensure that the prevention and treatment of perinatal mental health problems is one of the core business and reportable indicators in sub-city/city/nationally?
   - Explore: annual plans, monitoring and reporting systems, review meetings, etc
9. How is perinatal mental health integrated in routine maternal health services provision in health facilities or health systems or maternal health guidelines and strategies (at national level)?

*Explore*

- - *Planning of perinatal mental health*
  - *Model of integration (antenatal care, labour and childbirth, and postnatal care)*
  - *Staff capacity and awareness to detect and manage mental health issues*
  - *Client referral, mental health clinic in the same facility, etc*
  - *Mental health promotion and prevention vs mental health issues treatment*
  - *Screening tools*

1. What are the challenges to provide perinatal mental health care in health facilities/health systems?
   - *Explore: governance (accountability, stewardship), leadership, providers’ knowledge, providers’ mental health (self-care), staff availability, staff motivation, team spirit, infrastructure, client load, etc*
2. What could be done to improve perinatal mental health care in your sub-city/city/nationally?
   - Activities in the scope of responsibility of health facilities
   - Activities in next higher level/s of the health system

**Additional file 3B: In-depth interview guide for interviews at health facility-level (maternity staff, maternal health service coordinator, head of department or facility)**

***Background characteristics of key-informant***

| *Organisation* |  |
| --- | --- |
| *Position* |  |
| *Current profession* |  |
| *Previous role in health facilities (Y/N)* |  |
| *Service year in current position* |  |
| *Service year in the health system* |  |
| *Interview date and time* | *__dd/__mm__yy From __:__ am/pm* ***to*** *__:__am/pm* |

**Interview guide**

1. In your opinion what are the characteristics/domains of high-quality care for women at the time of labour and childbirth?
   - *Explore: infrastructure, human resource, client-provider relationships, mental health needs of women*
2. Sometimes women experience behaviours or conditions which are perceived as negative or disrespectful during childbirth in health facilities. From your experience, what kinds of such behaviours or conditions happen during childbirth in health facilities??
   - *Provide clear explanation of what mistreatment is when saturation of ideas is reached for this question*
3. It is reported that women are sometimes disrespected and even abused at the time of labour and childbirth. In your opinion, what factors contribute to this problem?
   - *Explore: providers’ knowledge and attitude, staff shortage, staff motivation, team spirit, infrastructure, client load, etc*
4. Would you please describe what you imagine under respectful maternity care?
   - *Provide clear explanation of what respectful maternity care is when saturation of ideas is reached for this question*
5. What do you think is the status of respectful maternity care in health facilities in Addis Ababa/Conakry?
   - *Explore: privacy, confidentiality, client-provider interaction, complaints on non-respectful care, etc*
6. How is your health facility actively promoting respectful maternity care? If it doesn’t, why not?
   - *Explore: ongoing initiatives, guidelines, IEC/BCC activities, observe maternity settings*
7. If yes in 6, what challenges do you encounter in promoting respectful maternity care in your health facility?
   - *Explore: providers knowledge and attitude, staff availability, staff motivation, team spirit, infrastructure, client load, promoting providers’ right, etc*
8. What measures are being taken to ensure that the prevention and treatment of perinatal mental health problems one of the core business and reportable indicators in your facility?
   - Explore: annual
9. How is perinatal mental health integrated in routine maternal health services provision in your facility?

*Explore*

- - *Planning of perinatal mental health*
  - *Model of integration (antenatal care, labour and childbirth, and postnatal care)*
  - *Focus on vulnerable groups*
  - *Staff capacity and awareness to detect and manage mental health issues*
  - *Client referral, mental health clinic in the same facility, etc*
  - *Mental health promotion and prevention vs mental health issues treatment*
  - *Screening tools*

1. What challenges do you encounter in providing maternal mental health care in your health facility?
   - *Explore: providers’ knowledge, providers’ mental health (self-care), staff availability, staff motivation, team spirit, infrastructure, client load, etc*
2. In your opinion, what could be done to improve maternal mental health care in health facilities?
   - Activities in the scope of responsibility of health facilities
   - Activities in next higher level/s of the health system
