# Supplementary material for: Is MIStreatment of women during facility-based childbirth an independent risk factor for POstpartum Depression in Ethiopia and Guinea? A mixed methods prospective study protocol—MISPOD study
Source: Reprod Health. 2024 Sep 4;21:129. doi: 10.1186/s12978-024-01850-w (PMC11375852; doi:10.1186/s12978-024-01850-w)
Supplement: Supplementary file 4 — Additional file 4: Interview guides for in-depth interviews with women. [file 12978_2024_1850_MOESM4_ESM.docx]

**Additional file 4: In-depth interview guide for interviews with women who experienced perinatal depression**

***Background characteristics of key-informant***

| *Sub-city* |  |
| --- | --- |
| *Questionnaire ID-from women’s survey* |  |
| *Date of birth* | *__dd/__mm__yy* |
| *Place of birth (write name of health facility otherwise home)* |  |
| *Interview date and time* | *__dd/__mm__yy From __:__ am/pm* ***to*** *__:__am/pm* |

**Interview guide**

1. What mental health tips and advice did you receive from health workers during your antenatal, childbirth or postpartum care?
   - *Explore: content of care, setting of care, etc*
2. Would you please tell me your experience since you have given birth?
   - *Explore: mental and physical recovery, sleep deprivation, medical conditions (mother and infant), etc*
3. What difficulties have you faced since giving birth? How have you been trying to solve these difficulties?
   - *Explore: health issues, including mental health, social problems, newborn/child care, intimate/family relationship-related issues, etc*
   - *Explore: role of partner, family members, social support, etc in managing difficulties*
4. How did these difficulties affect your and your baby’s health?
   - *Explore: care for baby, mother’s emotional status, etc*
5. How was the support you received from your partner, relatives, and friends (if any)?
   - *Explore: possible stigma the women might have experienced, loneliness,*
6. Did anyone from the health facilities where you sought care for pregnancy, childbirth, or postpartum care inquired for the difficulties you experienced?
   - *Explore: what support mechanisms did you get? Was it meaningful?*
7. How has been your experience with the health workers who tried to help you?
   - *Explore: humaneness, empathy, level and type of support*

Thank you again for your willingness and time to participate this interview today telling us about your experiences during pregnancy, childbirth, and the postpartum period. We would also like to ask you more about the mental health difficulties you experienced, the care and support you received, and what needs to be done to improve the experiences of women who pass through similar difficulties.

1. What would you have done differently if you were to provide care for women with postpartum depression?
